# Evolution of Multicellularity

**DOI:** 10.3390/genes12101532

**Published:** 2021-09-28

**Authors:** J. Mark Cock

**Affiliations:** CNRS, Sorbonne Université, UPMC University Paris 06, Algal Genetics Group, UMR 8227, Integrative Biology of Marine Models, Station Biologique de Roscoff, CS 90074, 29688 Roscoff, France; cock@sb-roscoff.fr

The emergence of multicellular organisms was, perhaps, the most spectacular of the major transitions during the evolutionary history of life on this planet [1,2]. Despite decades of research aimed at understanding this important transition, many questions remain unanswered. This Special Issue, “Evolution of Multicellularity”, which consists of reviews, an opinion piece and original research articles, highlights and addresses some of these questions. These articles look at several aspects of the evolution of multicellularity, ranging from the evolution of key molecular components to the emergence of cellular and organism-level features. They consider both early events at the transition between unicellularity and multicellularity and the subsequent complexification of multicellular organisms. Together, the articles present several novel observations and propose interesting new hypotheses aimed at stimulating future research in this area.

Research into the evolution of multicellularity tends to involve either studies that attempt to understand transitions from unicellularity to multicellularity or studies that focus on the subsequent step, corresponding to the emergence of complex multicellularity (i.e., the evolution of macroscopic organisms with multiple cell types, such as animals and land plants). Kin and Schaap [3] argue that this distinction is somewhat artificial and point out that, in fact, lineages may exhibit a continuum of different levels of complexity and that this continuum can provide useful material for the investigation of the evolutionary origins of multicellularity. One such lineage is the dictyostelid social amoebas. Analysis of this lineage supports a broad hypothesis that is probably relevant to diverse multicellular lineages [4,5,6,7]: that the adaptation of what the authors call “proto cell-types” to particular environmental conditions may have laid the foundations for the later emergence of distinct cell types in a multicellular decedent.

Extracellular matrices (ECMs) have played key roles in the evolution of multicellularity. They not only carry out the basic physical role of binding cells together but also influence development and morphology, protect cells from the external environment and represent an important a source of molecules involved in intercellular communication, all essential functions for a multicellular organism. Kloareg et al. [8] provide an overview of the diversity of ECMs across eukaryotes, the origins of these structures and the roles they have played in the emergence of multicellularity. The article focuses particularly on macroalgae, which, perhaps, represent the least well understood of the complex multicellular eukaryotic lineages. An important conclusion from this analysis is that very few elements of ECMs can be traced back to the last eukaryotic common ancestor, indicating that these structures have evolved independently in each of the major complex multicellular eukaryotic lineages.

Transcription-associated proteins (TAPs, i.e., transcription factors and other transcription-associated proteins) play a central role in mediating and modulating the expression of the genome in the different cell types of a multicellular organism. In this Special Issue, Petroll et al. [9] focus on the TAP complement of red algae, comparing unicellular and multicellular species. Analysis of gene family expansions identified TAP families that may have played important roles in key events during red algal evolution.

An opinion article by Patthy [10] focuses on the emergence of the molecular tool kit that accompanied the evolution of multicellularity in the Metazoa. The author observes that most of the multidomain proteins involved in cell–cell and cell–matrix interactions have evolved by exon shuffling, but this does not appear to be the case for transcription factors. The explanation proposed for this difference is that the transcription factors may have evolved earlier, possibly before the emergence of multicellularity, a suggestion that is in accordance with the idea of the emergence of “proto cell-types” (see above) as proposed by Kin and Schaap [3].

Finally, the article by Isaksson et al. [11] considers the transition from unicellularity to multicellularity from a novel perspective, focusing on the direct consequences of inherited features of the unicellular ancestor for multicellular growth rather than the adaptation of such features in a multicellular context. Based on mathematical modelling. they predict that the type of cell division inherited from the unicellular ancestor, budding or fission, will influence the rate of spread of beneficial mutations in the multicellular lineage and, consequently, its capacity to adapt and compete with other organisms for a niche. This study emphasizes that it is not only the mode of acquisition of multicellularity that is important but also the tempo, with lineages that can rapidly fix beneficial mutations being at an advantage.

In conclusion, this Special Issue brings together articles that address several of the important outstanding questions in multicellularity research and should be of interest to a broad audience interested in both the experimental and theoretical aspects of this exciting field of research.
